# Emergency surgical consultation for cancer patients: identifying the prognostic determinants of health

**DOI:** 10.1186/s12957-022-02694-z

**Published:** 2022-07-12

**Authors:** Kadhim Taqi, Diane Kim, Lily Yip, Charlotte Laane, Zeeshan Rana, Morad Hameed, Trevor Hamilton, Heather Stuart

**Affiliations:** 1 Department of Surgery - Division of General Surgery, University of British Columbia, 2103, 638 Beach Crescent, Vancouver, BC V6Z3H4 Canada; 2 University of British Columbia, Faculty of Medicine, 317–2194 Health Sciences Mall, Vancouver, BC V6T 1Z3 Canada; 3 Department of Surgery - Division of General Surgery, Vancouver General Hospital, 2775 Laurel Street, 11th Floor, Vancouver, BC V5Z 1M9 Canada

**Keywords:** Emergency cancer surgery, Surgical oncology, Prognostic determinants, Vulnerable, Acute care surgery

## Abstract

**Background:**

Patients with malignancy often require urgent surgical consultation for treatment or palliation of disease. The objective of this study is to explore the prognostic determinants affecting care in acute cancer-related surgical presentations and the effect on patient outcomes.

**Main body:**

This is a retrospective review of patients referred to the acute general surgery (ACS) service at a tertiary hospital for management of cancer-related problem from July 2017 to September 2018. Patient demographics, course in hospital, and survival were recorded. Multivariant logistic regression and Kaplan-Meier estimates were performed. One hundred eighty-nine patients were identified (53% female) with a mean age of 65.9 years. Forty-two patients (22%) were newly diagnosed with cancer on presentation, and 94 (50%) patients had metastatic disease. Cancer staging was completed in 84% of patients, and 65% had multidisciplinary team (MDT) assessment during their hospital stay. Surgery was performed on 90 (48%) patients, of which 31.2% was with palliative intent. Overall mortality was 56% with 30- and 60-day mortality of 15% and 22%, respectively. The adjusted odds ratio (OR) for a 60-day mortality was high in patients presenting with new cancer diagnosis (OR 3.18, 95% CI 1.18–9.02, *p*=0.03), metastatic disease (OR 5.11, 95% CI 2.03–12.85, *p*=0.001), or systemic therapy on presentation (OR 3.46, 95% CI 1.30–9.22, *p*=0.013).

**Conclusion:**

Emergency surgical referral is common in patients with malignancy. Surgical decision making can be challenging due to the heterogeneity of this population and their associated comorbidities. Optimizing prognostic determinants such as goal-directed palliative care, MDT discussions, and bridging to systemic therapy can improve patient outcomes.

## Background

Cancer is the leading cause of death in Canada with an estimated 225,800 new cancer diagnoses and 83,300 cancer-related deaths in 2020 [1]. A significant number of patients with malignancy are presented to the emergency department (ED) with acute symptoms [2]. In the USA, more than 4 million patients annually visit the ED for malignancy-related concerns, with up to 59% requiring admission [3–5]. Patients requiring emergency cancer operations have worse outcomes than those undergoing scheduled operations [6–10]. They are more likely to present with advanced stage and suffer higher morbidity and mortality [7–10]. Preoperative optimization can be challenging in this population due to factors such as poor nutritional status, comorbidities, and lack of cancer staging [5, 11–13]. There is limited data in this area as the heterogeneity of patients presenting with acute malignancy-related concerns makes it difficult to analyze outcomes on an individual basis. Therefore, there is a need to identify themes within patient care pathways that can be used to build infrastructure and enhance care for this population [9]. Themes can be categorized into determinants of health such as social, physical, and prognostic determinants. Social and physical determinants that influence outcomes have been described in the literature and include factors like income, ethnicity, or body mass index [14]. Prognostic determinants include assessments and investigations that predict disease burden and assist with developing treatment strategies [15]. The primary objective of this study is to identify prognostic determinants of health that predict outcomes for patients with acute cancer-related surgical presentations and provide guidelines for optimizing care pathways.

## Main text

### Methods

A retrospective study was performed of patients assessed by the inpatient acute care surgery (ACS) service for the management of any cancer-related surgical problem at Vancouver General Hospital in British Columbia. The study received an approval by the research ethics board. Patients were identified from a REDcap database® of all patients referred to ACS between July 2017 and September 2018 and were included if they were found to have a cancer-related problem at any point during their admission. Time to intervention including surgery, endoscopy, and interventional radiology (IR) was calculated from the day of admission, while time to adjuvant therapy was calculated from the day of discharge. A multidisciplinary team (MDT) was defined as the inclusion of more than one hospital-based team during a patient’s hospital stay. Surgery with palliative intent was defined as a surgical intervention performed in the setting of non-curable disease to provide symptom relief and improve the quality of life. Prognostic determinants of health were defined as factors that predict the outcome of a disease process [15, 16]. A comparison between surgery versus no surgery was made using Student’s *t* test. Overall, a 30- and 60-day mortality were calculated from the day of admission to hospital and were compared using Student’s *t* test. Kaplan-Meier analysis was performed comparing overall survival (OS) between patients who underwent surgery and non-surgical patients. A multivariate Cox hazard model was performed using multiple covariates including age, sex, primary cancer site, distant metastasis, date of diagnosis, systemic therapy on presentation, and cancer therapy within 12 weeks of discharge from hospital. Multivariate analysis was performed using logistic regression. A *p* value of <0.05 was statistically significant.

## Results

One hundred eighty-nine patients were referred to ACS for malignancy-related surgical problems, comprising of 11% of all general surgery consults (*n*=1725). The mean age was 65.9 ± 14.5 years with 53% female. Forty-two patients (22.2%) received their first diagnosis of cancer during this presentation, and 33.4% of these patients (14/42) had metastatic disease. Of the patients with an established malignant diagnosis (77.8%), 54.4% (80/147) had metastatic disease at the time of referral to ACS. The most frequent primary sites of malignancy were lower gastrointestinal (LGI) (colon and rectum) (51.3%), non-visceral (skin, breast, sarcoma, and lymphoma) (15.3%), and upper gastrointestinal (UGI) (esophageal, gastric, small bowel, and pancreas) (13.8%). The most common reasons for referral were bowel obstruction, post-operative complications (from a separate hospital admission), and admission for urgent staging investigations or interventions. Fifty-five patients (29.1%) were on systemic therapy on presentation (i.e., chemotherapy, immunotherapy). Table 1 shows the demographics for cancer related referrals.

**Table 1:** Demographics of patients that underwent surgery compared to no surgery

Characteristic	All patients ***N*** (%)	Surgery ***N*** (%)	No surgery ***N*** (%)	***P*** value
Total patients	189	90 (47.6)	99 (52.4)	
Age in years, mean±SD	65.9±14.5	67.5±13.7	64.5±15.1	0.158
Female	101 (53.4)	45 (50)	56 (56.6)	0.817
Type of cancer				
Lower GI	97 (51.3)	54 (60)	43 (43.4)	
Upper GI	26 (13.8)	10 (11.1)	16 (16.2)	
Non-visceral^a^	29 (15.3)	12 (13.3)	17 (17.2)	0.051
Hematological	11 (5.8)	7 (7.8)	4 (4)	
Others^b^	26 (13.8)	7 (7.8)	19 (19.2)	
New cancer diagnosis	42 (22.2)	27 (30)	15 (15.2)	**0.014**
Metastatic disease	94 (49.7)	35 (38.9)	59 (59.6)	**0.004**
Systemic therapy^c^	55 (29.1)	14 (15.6)	41 (41.4)	**<0.001**
Adjuvant therapy^d^	70 (37)	37 (41.1)	33 (33.3)	0.269
Radiation therapy	2 (1.1)	0	2 (2)	-
Mortality	105 (55.6)	48 (53.3)	57 (57.6)	0.558
30-day mortality	28 (14.8)	6 (6.7)	22 (22.2)	**0.003**
60-day mortality	41 (21.7)	14 (15.6)	27 (27.3)	0.051

*SD* standard deviation

^a^Non-visceral: skin, breast, and sarcoma

^b^GYN, urological, ENT, and lung

^c^Active cancer therapy including chemotherapy, immunotherapy, or targeted therapy

^d^Cancer therapy within 12 weeks postoperatively

**Fig. 1:**
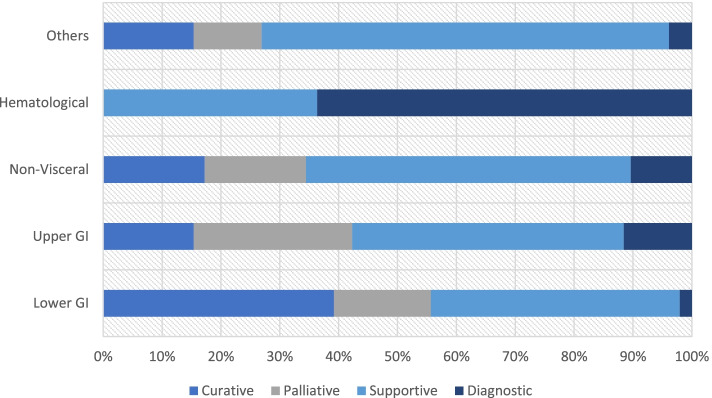
The goals of care and the management approach based on the primary cancer site. Curative approach was defined as undergoing surgery with a goal of cure. Palliative approach was defined as surgical or conservative management with the goal of symptom control with a non curative intention. Supportive management indicated that patients underwent non-operative management of a cancer related problem or complication. Diagnostic approach mean that patients were admitted for cancer diagnosis or staging

Of the 189 referred patients, 99/189 (52.4%) received non-operative management, 28/189 (14.8%) underwent surgery with palliative intent, 49/189 (25.9%) underwent surgery with curative intent, and 13 underwent surgery for diagnostic purposes. Thirty-five patients had an endoscopic procedure (e.g., endoscopy, endoscopic ultrasound, stent placement), and 25 had a procedure performed by IR (e.g., percutaneous biopsy, drain placement, aspiration). Complete staging with computed tomography (CT) of the chest, abdomen, and pelvis was performed in 84.4% (76/90) of the patients who underwent surgery. Figure 1 shows the sites of the primary malignancy and the goals of care with intervention performed.

The mean length of hospital stay was 12.2 ± 15.4 day. The MDT approach was performed in 65% of the patients. The average waiting time to surgery was 3.2± 4.4 days from admission, while time to endoscopy and IR was 2.8± 6.7 days and 3.3± 3.8 days, respectively. Most patients were discharged home (81.2%) (Table 2).

**Table 2:** In-hospital care and disposition of the patient population

Mean length of stay (days)	12.2 ± 15.4	***N*** (%)
**Origin of consult**^a^	ED	135 (71.4)
	Inpatient	26 (13.8)
	Direct admission	28 (14.8)
**Consulted services**	Gastroenterology	43 (22.8)
	Intervention radiology	25 (13.2)
	Hematology	19 (10.1)
	Internal medicine	18 (9.5)
	Palliative services	17 (9)
**In-hospital interventions**	Surgery	90 (47.6)
	Endoscopy	35 (18.5)
	Intervention radiology	25 (13.2)
**Reason for surgery**	Curative	49 (54.4)
	Palliative	28 (31.2)
	Diagnostic	13 (14.4)
**Disposition**	Home	155 (82)
	In-hospital mortality	17 (9)
	Rehabilitation	5 (2.6)
	Transfer	12 (6.4)

^a^Origin of consult is defined as (1) ED if the patient has been referred by an emergency room physician, (2) inpatient if the patient was admitted under a service other than general surgery was referred to general surgery during their hospital admission, and (3) direct admission if the patient was referred to the inpatient ACS service directly from an outpatient physician (e.g., family doctor or other specialists’ physician)

**Fig. 2:**
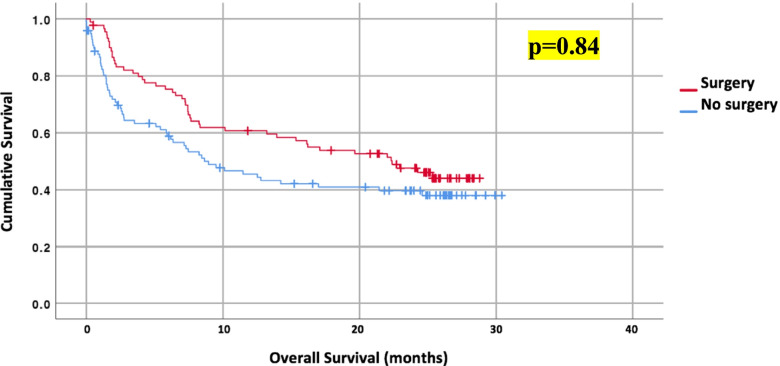
A Kaplan-Meier curve of overall survival (OS) between the surgery and non-surgery groups. Patients undergoing surgery had higher OS compared to no surgery (22.4 vs. 8.6 months, *p*=0.84)

Overall mortality was 55.6% with 30-day mortality and 60-day mortality rates of 14.8% and 21.7%, respectively. Compared to patients managed non-surgically, patients who underwent surgery had lower 30-day (6.7% vs. 22.2%, *p*<0.05) and 60-day mortality (15.6 vs. 27.3%, *p*=0.051). The median survival was 15.4 months for the entire population with a clinical trend towards a longer survival in patients who underwent surgery compared to non-surgical management (surgery vs. no surgery, 22.4 vs. 8.6 months, *p*=0.84). Patients with early MDT involvement during their presentation were found to have lower 30-day mortality (OR 0.61, 95% CI 0.50–0.73, *p*=0.001). Sub-analysis was performed comparing patients undergoing palliative surgery compared to non-operative management. The 30-day mortality was lower in patients undergoing surgery with palliative intent (10.7 vs. 22.2%, *p*<0.05); however, there was no difference in the 60-day mortality (21.4% vs. 27.3%, *p*=0.19) or overall survival (palliative surgery vs. no surgery, 9.7 vs. 11.6 months, *p*=0.39). Figure 2 illustrates a Kaplan-Meier curve of the overall survival (OS) between the surgery and non-surgery groups. Table 3 shows the adjusted hazard ratios (HR) for the overall mortality of the population. The odds ratio (OR) for a 30- and 60-day mortality in patients undergoing surgery was 0.29 (95% CI 0.10–0.81, *p*=0.019) and 0.63 (95% CI 0.28–1.43, *p*=0.270), respectively. New cancer diagnosis (OR 3.18, 95% CI 1.12–9.02, *p*=0.030), systemic therapy (OR 3.46, 95% CI 1.3–9.22, *p*=0.013), and metastatic disease on presentation (OR 5.11, 95% CI 2.03–12.85, *p*=0.001) were associated with higher odds of a 60-day mortality. Table 4 shows the adjusted OR for a 30- and 60-day mortality.

**Table 3:** Adjusted hazard ratios for overall mortality in oncological patients with acute surgical issues

Variable	Hazard ratio	95% confidence interval	***P*** value
Age	1.01	0.99–1.02	0.228
Female	0.82	0.53–1.27	0.375
Surgery	0.78	0.51–1.20	0.261
GI cancer	0.97	0.63–1.49	0.876
New cancer diagnosis	1.63	0.93–2.85	0.090
Metastatic disease	5.59	3.37–9.26	**<0.001**
Systemic therapy	1.57	0.97–2.57	0.069
Adjuvant therapy^a^	0.40	0.25–0.65	**<0.001**

^a^Cancer therapy within 12 weeks postoperatively

**Table 4:** Adjusted odds ratios (OR) for a 30- and 60-day mortality

Variable	30-day mortality			60-day mortality		
	OR	95% CI	***P*** value	OR	95% CI	***P*** value
Age	1.04	1.00–1.08	0.031	1.04	1.01–1.07	**0.015**
Female	0.70	0.27–1.81	0.466	0.46	0.20–1.06	0.068
GI cancer	1.10	0.43–2.80	0.841	0.79	0.35–1.77	0.570
New cancer diagnosis	0.32	0.09–1.08	0.067	3.18	1.12–9.02	**0.030**
Metastatic disease	4.63	1.56–13.68	0.006	5.11	2.03–12.85	**0.001**
Systemic therapy	2.61	0.85–8.01	0.093	3.46	1.30–9.22	**0.013**
Surgery	0.29	0.10–0.81	0.019	0.63	0.28–1.43	0.270

## Discussion

### Establishing prognostic determinants

**Fig. 3:**
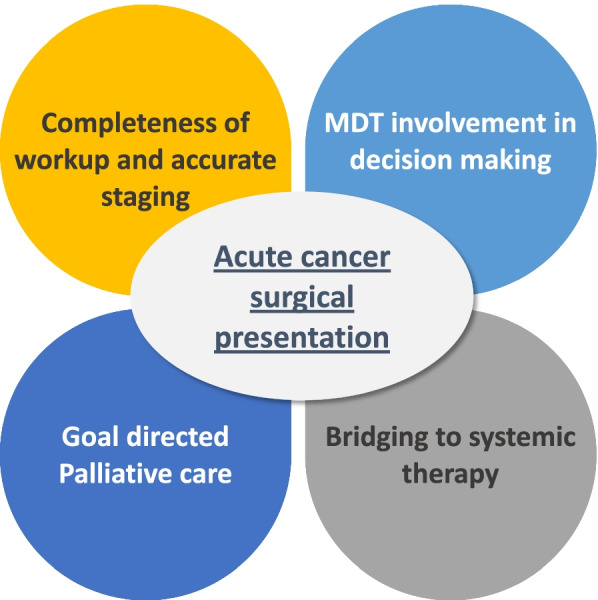
The pillars of prognostic determinants of cancer in the context of acute cancer surgical presentation

Patients with acute oncologic surgical presentations are a vulnerable population that require thoughtful management strategies to optimize the risk benefit ratio of treatment. The determinants that influence their treatments are complex and multifaceted, so by enhancing our understanding of these factors, there is a potential to improve outcomes. Prognostic determinants are modifiable factors that contribute to the understanding of an individual’s burden of disease and when identified can contribute to the selection of effective management strategies [14–17]. This study highlights several factors including new cancer diagnosis, the presence of metastatic disease, and systemic therapy that aid in establishing patient prognosis. By identifying prognostic factors related to disease, theme-based prognostic determinants can be developed that can enhance patient experience and outcomes. The themes of prognostic determinants identified in this paper include completeness of work up and accurate staging, multidisciplinary assessment, goal-directed palliative care involvement, and bridging to systemic therapy (Fig. 3).

### Completeness of workup and accurate staging

An important component of oncologic care is staging of disease, including tissue diagnosis and appropriate imaging. This can be challenging in the emergency setting because of the acuity of presentation. Although the initial goal in an urgent setting is to ensure patient stability, the type of cancer and the extent of malignancy are essential in determining prognosis and guiding medical and surgical management [10, 18]. One example depending on histologic diagnosis would be treatment of bleeding tumors with radiation rather than surgical resection to avoid wounding healing complications and incomplete resection [19]. Studies show that up to 30% of patients with emergency cancer presentations have an unknown stage on presentation, and around 18% of patients with colorectal cancer undergo surgery without complete staging [20, 21]. In our study, over one third of patients with a new diagnosis of cancer had metastatic disease. As metastatic disease is a predictor of overall mortality (HR 5.59, *p*<0.001), it is important to establish this early in a patients’ care pathway to guide treatment appropriately. For example, in a patient with known lung metastases from colon cancer presenting with a malignant bowel obstruction, a diverting ostomy may be favored over an extensive bowel resection. Therefore, the completeness of workup is an important prognostic determinant in the setting of surgical care as it can influence operative and non-operative planning [10, 22].

### Multidisciplinary assessment

Patients with malignancy have diverse care needs that require multidisciplinary assessment to ensure a comprehensive care plan is developed. Assessments can include, but are not limited to, surgical and medical consultants, symptom management specialists, nursing, and allied health teams. The involvement of these members is important for all patients but becomes essential for patients with emergency presentations. Bosscher et al. showed that MDT evaluation of patients with acute cancer presentations improved accurate assessment of physical status and prevented overtreatment in advanced stages [9, 19]. Early advanced practice nursing involvement in the care of elderly patients with cancer who received surgical intervention improved survival in this group by an average of seven months. As well, the need for emergency care was lower in patients receiving MDT treatment within a year of cancer diagnosis (OR=0.87) [23,24]. In our study, 65% of the patients had MDT assessment during their stay comprised largely by gastroenterology and interventional radiology. Patients with MDT involvement were found to have lower 30-day mortality (OR 0.61, 95% CI 0.50–0.73, *p*=0.001). However, attributing this improvement in survival with MDT assessment can be difficult due to the heterogeneity of the population. This finding, in combination with previous studies noting a survival advantage with MDT care, supports the inclusion of the MDT assessment as a prognostic determinant. Input from appropriate specialists on nuances of care outside the surgical realm of practice such as interventional techniques, available systemic options, and health optimization is essential for enhancing prognosis.

At our institution, an ACS model was developed to address the acute general surgery demands of a tertiary referral center. By recognizing the unique needs of oncology patients within this larger population, infrastructure for care has been developed as part of an integrated practice unit (IPU). An emergency surgical oncology IPU was initiated by mapping care pathways to identify rate limiting steps or areas for improvement (diagnostic, interventional, or consultant based). An example of MDT implementation based on IPU mapping involves an oncology care provider attending daily surgical handover rounds to provide recommendation and feedback for patients presenting with acute oncologic concerns. MDT involvement allows for more accurate prognostication which may circumvent unnecessary invasive procedures or facilitate access to appropriate systemic therapy. Hence, it becomes a prognostic determinant with the potential to improve patient outcomes and should be considered for all emergency surgical oncology presentations.

### Goal-directed palliative care

Involvement of palliative care teams and focus on symptom management can prolong life while improving quality [25–28]. Major surgical facilities report consultations for surgical palliation between 18 and 40% [9, 29]. In some centres, surgery with palliative intent accounts for up to one third of emergency surgeries in cancer patients [12, 29–31]. One approach to mitigating a surgical risk is through the “palliative triangle” as described by Miner et al. [25]. The palliative triangle is a communication strategy through which the potential for reaching treatment goals, durability of the procedure, and postoperative complications are discussed with the patients and their families preoperatively to create realistic expectations and guide the decision-making process. Using this method, Miner et al. noted that only 47% of the patients for which palliative surgery was discussed underwent a procedure. This suggests that with appropriate insight into the proposed value and purpose of surgery, patients may choose alternative strategies to achieve personal goals. The early involvement of a goal-directed palliative care teams allow patients to explore options for optimizing symptom control and quality of life which potentially provide a survival advantage [32, 33]. The ENABLE III trial in 2015 showed that patients with early palliative care involvement (within 30–60 days of diagnosis) were shown to have an improved 1-year survival (63%) compared to the delayed group (48%, 3 months after diagnosis) [34]. As an example, in our population, 31.2% of surgeries were performed with palliative intent. Most patients who underwent palliative surgery were discharged home (93%), and many went on to have systemic therapy (25%) postoperatively. As reception of adjuvant therapy is associated with improved overall survival, factors that facilitate adjuvant therapy would be considered a prognostic determinant. Moreover, patients whom underwent goal-directed palliative surgical therapy had lower 30-day mortality compared to nonsurgical patients (10.7 vs. 22.2%, *p*<0.05). As such, goal-directed palliative care is an important prognostic determinant with prior literature suggesting that early involvement may provide maximum benefit to patients.

### Facilitating systemic therapy

Timely initiation of adjuvant chemotherapy is a positive predictor of outcomes in patients with cancer [30]. In patients with emergency cancer presentation, the majority are potential candidates for systemic therapy [7–10]. The need for urgent surgical intervention may lead to an interruption of systemic therapy or delay in initiation, both of which can impact overall and cancer specific survival [35]. Treatment delay can also lead to negative effect on local control rates, functional outcomes, complications from disease progression, and quality of life [35]. In our population, the ability to receive adjuvant systemic therapy was associated with a reduced risk of mortality (HR 0.40, 95% CI 0.25–0.65, *p*<0.001)). A study by Heyler et al. in 2007 examined the role of palliative surgery in patients with malignant bowel obstruction and showed a survival benefit in patients undergoing surgery as a bridge to systemic therapy compared to surgery alone or no surgery (10.3 versus 0.3 versus 2.7 months, respectively) [36]. This highlights the importance of reception systemic therapy as a prognostic determinant and encourages surgeons to explore options for facilitating this through individual care plans.

This study is limited by its retrospective nature for which comprehensive patient demographics and treatment course were difficult to capture. For example, post-operative complications were not available for collection, which represent an important aspect on evaluating surgical management. The heterogeneity of the study population presents a challenge when applying generalized conclusions to an individual patients’ specific cancer diagnosis. Larger sample sizes and prospective follow-up are necessary to further clarify the influence of MDT dynamics and oncology-specific pathways on clinical and patient-reported outcomes.

## Conclusion

Surgical decision making for patients presenting malignancy-related emergencies is challenging. There are many factors that determine patient outcomes including social, physical, and prognostic determinants. By identifying prognostic determinants of health such as disease staging, early MDT involvement, and palliative care assessment, patient care pathways can be initiated that have the potential to improve outcomes. The development of an emergency surgical oncology care pathway may help to establish appropriate treatment goals, minimize waiting times, and improve patient experience.

## Data Availability

The datasets used and/or analyzed during the current study are available from the corresponding author on reasonable request.

## Abbreviations

ED: Emergency department
ACS: Acute care surgery
IR: Interventional radiology
MDT: Multidisciplinary team
OS: Overall survival
LGI: Lower gastrointestinal
UGI: Upper gastrointestinal
CT: Computed tomography
HR: Hazard ratio
OR: Odds ratio
IPU: Integrated practice unit
